# Unveiling molecular mechanisms of pepper resistance to *Phytophthora capsici* through grafting using iTRAQ-based proteomic analysis

**DOI:** 10.1038/s41598-024-55596-3

**Published:** 2024-02-27

**Authors:** Fengyan Shi, Xi Zhang, Zhidan Wang, Xiuxue Wang, Chunlei Zou

**Affiliations:** 1 Vegetable Research Institute, Liaoning Academy of Agricultural Sciences, 84 Dongling Road, Shenhe District, Shenyang, 110161 China; 2 College of Horticulture, Shenyang Agricultural University, 120 Dongling Road, Shenhe District, Shenyang, 110866 China

**Keywords:** Differentially expressed proteins, Graft, iTRAQ, Pepper, *Phytophthora capsici*, Molecular biology, Plant sciences

## Abstract

*Phytophthora* blight severely threatens global pepper production. Grafting bolsters plant disease resistance, but the underlying molecular mechanisms remain unclear. In this study, we used *P. capsici-*resistant strain ‘ZCM334’ and susceptible strain ‘Early Calwonder’ for grafting. Compared to self-rooted ‘Early Calwonder’ plants, ‘ZCM334’ grafts exhibited delayed disease onset, elevated resistance, and reduced leaf cell damage, showcasing the potential of grafting in enhancing pepper resistance to *P. capsici*. Proteomic analysis via the iTRAQ technology unveiled 478 and 349 differentially expressed proteins (DEPs) in the leaves and roots, respectively, between the grafts and self-rooted plants. These DEPs were linked to metabolism and cellular processes, stimulus responses, and catalytic activity and were significantly enriched in the biosynthesis of secondary metabolites, carbon fixation in photosynthetic organizations, and pyruvate metabolism pathways. Twelve DEPs exhibiting consistent expression trends in both leaves and roots, including seven related to *P. capsici* resistance, were screened. qRT-PCR analysis confirmed a significant correlation between the protein and transcript levels of DEPs after *P. capsici* inoculation. This study highlights the molecular mechanisms whereby grafting enhances pepper resistance to *Phytophthora* blight. Identification of key genes provides a foundation for studying the regulatory network governing the resistance of pepper to *P. capsici.*

## Introduction

Pepper (*Capsicum annuum* L.) is an economically and socially important crop with a high economic value^1^. It is an important source of food colorants, medicinal compounds, spices, and vegetables and is widely cultivated in most countries worldwide^2^. In China, the planting area of pepper is 2.1 million hectares, with an annual output value of more than 40 billion dollars^3^. Pepper is rich in minerals and vitamins and has high nutritional value^4^. However, peppers are easily damaged by pests and diseases, especially *Phytophthora capsici* (*P. capsici*)^5^. *P. capsici* is a highly destructive soil-borne pathogen in pepper production that can lead to a decline of over 50% in pepper production or even complete crop failure in severe cases. It can infect various tissues of pepper plants, including the roots, stems, leaves, flowers, and fruits, causing the entire plant to wilt or even die^6^. Notably, this pathogen can harm a wide array of other crops, including cucumber, eggplant, green bean, pea, pumpkin, tomato, and watermelon^6–8^. Presently, the control of *P. capsici* largely depends on the application of chemical fungicides and cultivation practices, such as crop rotation, irrigation management, and soil solarization^9^.

Grafting is an important cultivation method for preventing soil-borne diseases and overcoming obstacles to continuous cropping in vegetable production^10^. Compared with biotechnology and breeding plans over many years, this technology is more convenient, simple, and environment friendly. Grafting of sensitive commercial varieties onto resistant rootstocks can effectively reduce the adverse effects of biotic and abiotic stresses on plants^11^. Therefore, the grafting technology is widely used in economically important vegetables, such as cucumber and tomato^1^. Grafting peppers onto resistant or partially resistant rootstocks can effectively reduce diseases^11^. For instance, grafted peppers exhibit high resistance to *Phytophthora* blight and bacterial wilt without showing any change in yield and fruit quality^12^. The proper combination of rootstock and scions can inhibit the infection and proliferation of nematodes and increase pepper yield^13^. However, research on the molecular mechanism of grafting to improve pepper disease resistance and the identification of related disease resistance genes remains limited.

Proteins are the final products of most genes that directly regulate plant metabolism and biological processes. The relative protein levels may differ owing to the role of different post-transcriptional regulatory mechanisms^14^. The comparative analysis method of differential proteomics can help in determining changes in protein levels to identify the proteins responsible for regulating the metabolic pathways involved in plant growth and development^15^. Although two-dimensional gel electrophoresis (2-DE) is the standard method for proteome research, it has some limitations, such as few repetitions and low sensitivity of protein detection^16^. To overcome these limitations, a new quantitative method, called isobaric tags for relative and absolute quantification (iTRAQ), has been developed, which can accurately and effectively quantify and compare protein expression in different samples^17^. Comparative proteomic analysis based on the iTRAQ technology has become an important tool for exploring the mechanism of action of fungicides against *P. capsici*. He et al. used iTRAQ-based proteomic analysis to reveal the inhibitory effect of 1,6-O,O-diacetylbritannilactone on *P. capsici*^18^. Mei et al. explored the antifungal molecular mechanism of benzothiazole against *P. capsici* using iTRAQ-based proteomic analysis and joint transcriptome analysis^19^. Wang et al. used the iTRAQ technology to analyze the protein expression of *P. capsici* treated with cinnamaldehyde^20^. They found that cinnamaldehyde inhibited the growth of *P. capsici* by interfering with its metabolic stability, providing a basis for exploring the molecular mechanism of the fungicide cinnamaldehyde on *P. capsici*. Pang et al. used the iTRAQ technology to analyze the proteome of *P. capsici* treated with piriformis, identified the targets of the fungicide and potential biomarker candidates, and revealed that proteomics is a useful tool for determining the action mechanism of new fungicides and evaluating the tolerance of pathogens to environmental changes^21^. Cai et al. explored the mode of action of the new fungicide SYP-14288 against *P. capsici* using proteome sequence analysis, providing a reference for the application of SYP-14288^22^. These observations indicate that proteomic analysis is an important means of obtaining information on specific biological reactions and exploring protein expression.

In this study, we aimed to identify the candidate proteins associated with improvement in the resistance of pepper to *Phytophthora* blight through grafting and further explore the defense mechanism of grafted pepper against *Phytophthora* infestation. To this end, we used the iTRAQ technology to screen and identify differentially expressed proteins from different resistant rootstocks.

This study utilized the *P. capsici*-immune strain ‘ZCM334’ and the susceptible strain ‘Early Calwonder’ as rootstocks, while ‘Early Calwonder’ served as the scion for grafting. The leaves and roots of ‘ZCM334’ grafted plants and ‘Early Calwonder’ self-rooted plants were sequenced based on iTRAQ, enabling the identification of differentially expressed proteins (DEPs). Subsequently, the significantly enriched metabolic pathways related to *P. capsici* were obtained. The expression characteristics of key DEPs in leaves and roots of ‘ZCM334’ grafted and ‘Early Calwonder’ self-root grafted plants at different stages following *P. capsici* infection were analyzed via qRT-PCR. The results of this study lay the foundation for exploring the genetic and molecular mechanisms underlying pepper’s resistance to *Phytophthora* blight.

## Results

### Phenotypic characteristics of ‘ZCM334’ grafted plants and ‘Early Calwonder’ self-rooted grafted plants

**Figure 1:**
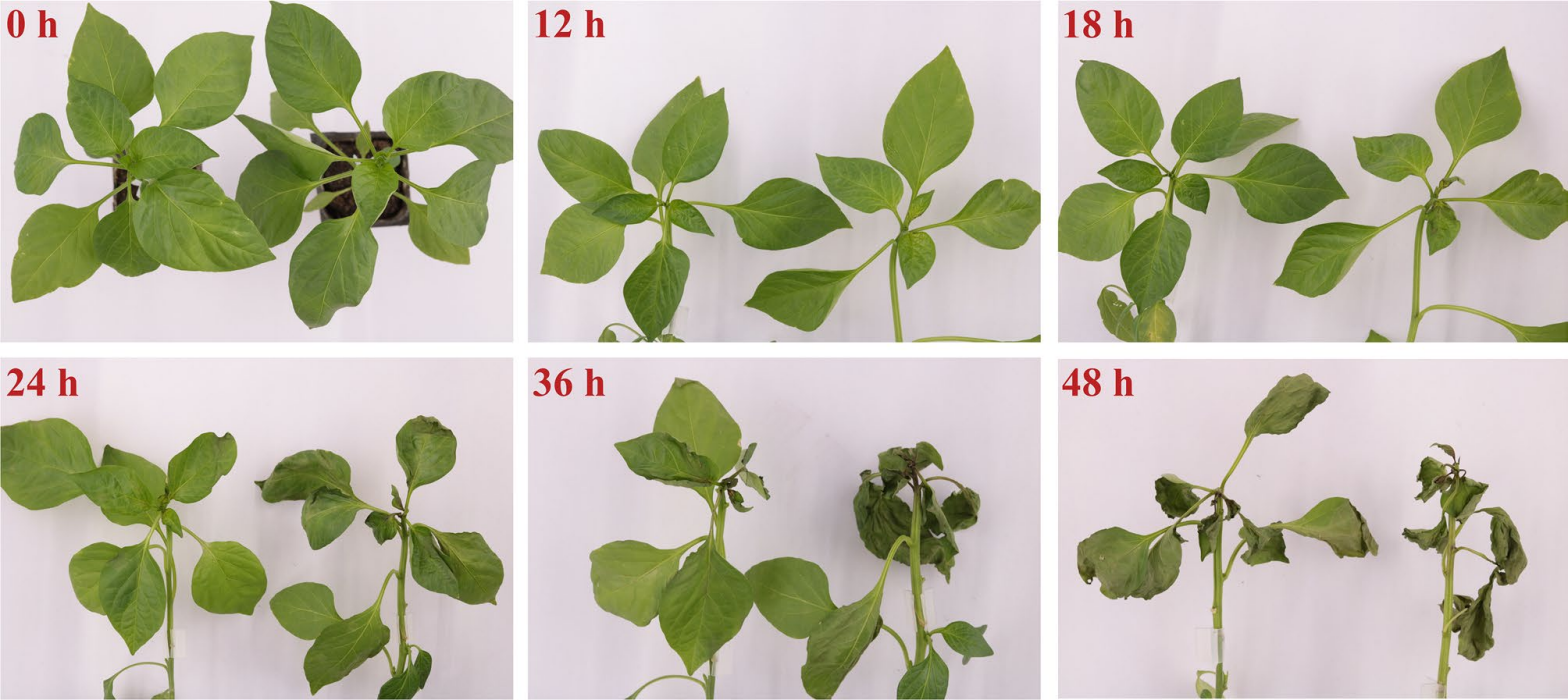
Observation of leaf phenotypes in ‘ZCM334’ grafted plants (left) and ‘Early Calwonder’ self-rooted grafted plants (right) inoculated with *Phytophthora capsici* at different stages.

**Figure 2:**
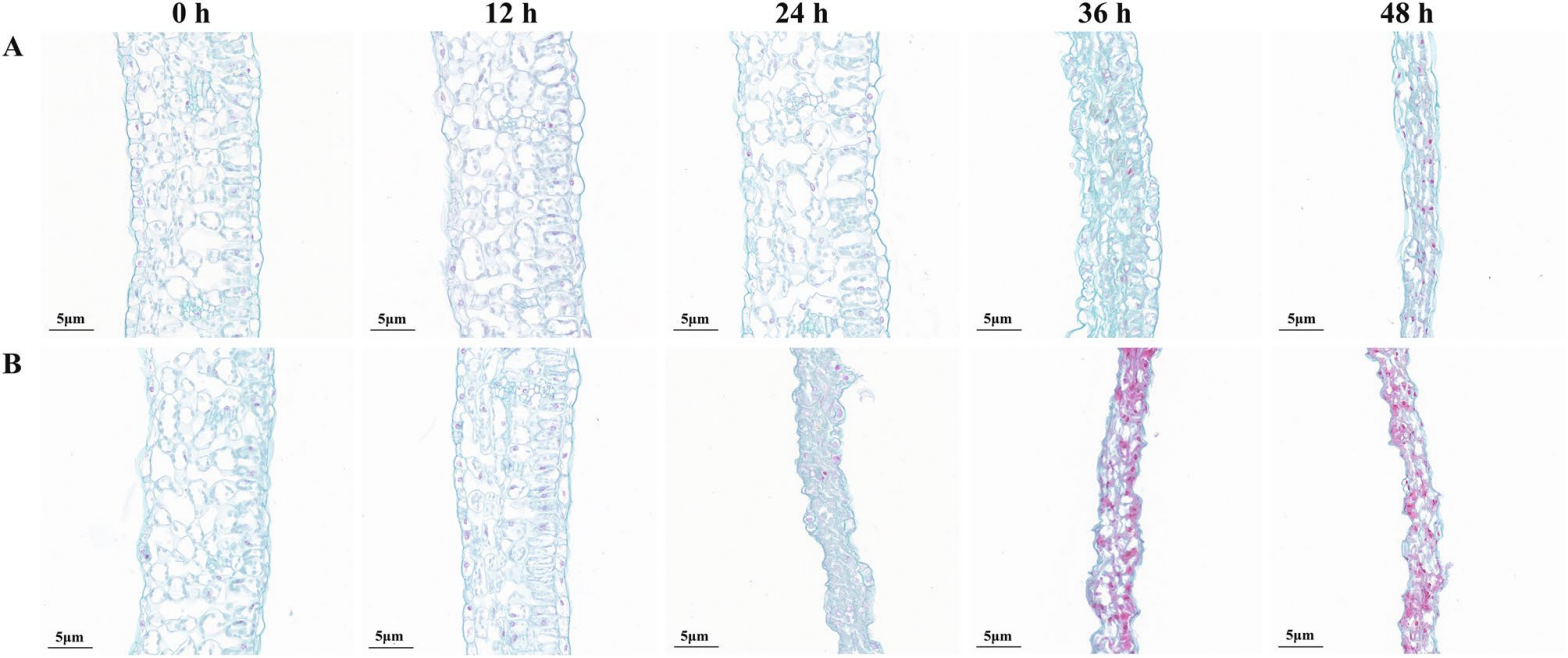
Observation of paraffin sections of leaves inoculated with *Phytophthora capsici* at different stages (0, 12, 24, 36, and 48 h) in ‘ZCM334’ grafted plants (**A**) and ‘Early Calwonder’ self-rooted grafted plants (**B**).

Through the phenotypic observation of ‘ZCM334’ grafted plants and ‘Early Calwonder’ self-rooted grafted plants at different stages after inoculation with *P. capsici*, we found that with the extension of inoculation time, the severity of the disease in the plant increased, and the plant leaves gradually withered after water loss. Compared with ‘Early Calwonder’ self-rooted grafted plants, ‘ZCM334’ grafted plants displayed delayed disease onset and robust resistance (Fig. 1). Paraffin section analysis revealed that following *P. capsici* inoculation, the palisade tissue, spongy tissue, and epidermal cells of the leaves of ‘Early Calwonder’ self-rooted grafted plants gradually atrophied, with a greater degree of deterioration than that observed in ‘ZCM334’ grafted plants (Fig. 2). At the 12th hour after *P. capsici* inoculation, the leaves of ‘ZCM334’ grafted plants remained unchanged, while the leaves at the growth point of ‘Early Calwonder’ self-rooted grafted plants turned black, indicating successful *P. capsici* infection. Subsequently, employing the iTRAQ technology, proteome sequencing was performed on the roots and leaves of ‘ZCM334’ grafted plants and ‘Early Calwonder’ self-rooted grafted plants after 12 h of inoculation.

### Basic information of total protein identification

In total, 368,895 spectra were obtained from the mass spectrometry experiment. After analysis using the Mascot software, 44,552 spectra, including 36,693 unique spectra, were matched. In total, 3410 proteins and 11,766 unique peptide segments were identified (Fig. S1A). The distribution of unique peptides is shown in Fig. S1B. Most of the identified proteins contained fewer than six peptides, and more than 32% of the proteins were mapped to one unique peptide. The protein mass distribution is shown in Fig. S1C. In the wide molecular weight range of 10–100 kDa, the coverage was very high (4–16% of the total protein in each molecular group). In terms of the distribution of peptide sequence coverage, 0–25% peptide coverage accounted for 87.18% (2973) of the total protein, whereas > 25% peptide coverage only accounted for 12.82% (437) of the total protein (Fig. S1D).

### Identification of DEPs

**Figure 3:**
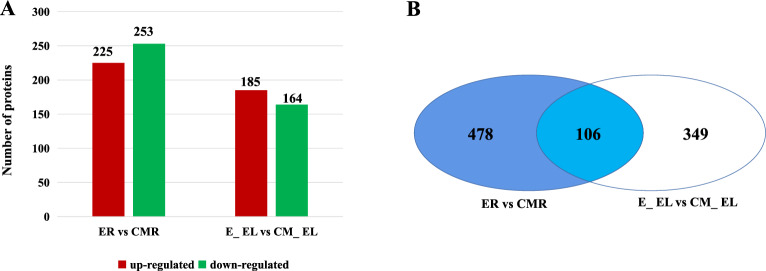
Statistics of differentially expressed proteins. (**A**) Number of upregulated and downregulated proteins identified in ER versus CMR and E_EL versus CM_EL. (**B**) Venn diagram displaying the number of differentially expressed proteins in ER versus CMR and E_EL versus CM_EL. ER, roots of ‘Early Calwonder’ self-rooted grafted plants; CMR, roots of ‘ZCM334’ grafted plants; E_EL, leaves of ‘Early Calwonder’ self-rooted grafted plants; CM_EL, leaves of ‘ZCM334’ grafted plants.

To ensure the accuracy of the identification of DEPs, iTRAQ was utilized with specific restrictions—a difference ratio > 1.2-fold and a *p*-value < 0.05. The comparative analysis of protein expression of the roots of the ‘Early Calwonder’ self-rooted grafted plants (ER) and the roots of the ‘ZCM334’ grafted plants (CMR) yielded 478 DEPs, including 225 upregulated and 253 downregulated DEPs in CMR (Fig. 3A, Table S1). Similarly, when comparing protein expression in the leaves of ‘Early Calwonder’ self-rooted grafted plants (E_EL) and those of ‘ZCM334’ grafted plants (CM_EL), 349 DEPs were identified, with 185 upregulated and 164 downregulated DEPs in CM_EL (Fig. 3A, Table S1). Remarkably, an intersection of DEPs was found in ER versus CMR and E_EL versus CM_EL, resulting in 106 commonly shared DEPs, encompassing 58 downregulated and 48 upregulated DEPs in CMR compared with those in ER and 56 downregulated and 60 upregulated DEPs in CM_EL compared with those in E_EL (Fig. 3B). In ER versus CMR and E_EL versus CM_EL, 81 DEPs exhibited consistent expression patterns in both the roots and leaves of ‘ZCM334’ grafted and ‘Early Calwonder’ self-rooted grafted plants, including 43 downregulated proteins and 38 upregulated proteins in CMR and CM_ EL.

### DEPs related to *P. capsici*

Understanding the basic processes and key genes related to *P. capsici* may provide important clues for exploring the molecular mechanisms underlying pepper resistance to *Phytophthora* blight. In the comparative proteome analysis of ER and CMR, some proteins related to *P. capsici* were identified, as shown in Table 1. These included the proteins downregulated in CMR, including one polygalacturonase CA03g35150, one leucine-rich repeat protein CA02g23570, one disease resistance response protein CA10g05650, one polyphenol oxidase CA02g15780, two cysteine proteases, four pathogenesis-related proteins, six β-1,3-glucanases, and eight peroxidase-like proteins, and the proteins upregulated in CMR, including one late blight resistance protein CA11g02470, one pathogen-related protein CA04g13070, two ascorbate peroxidase, and three aquaporins. In addition, two pectin methylesterases, two peptidyl-prolyl cis–trans isomerases, and four glutathione S-transferases were differentially expressed between ER and CMR.

**Table 1:** Identification of *Phytophthora capsici* resistance-related differentially expressed proteins between ER and CMR.

DEPs_ID	Annotation	Mean_Ratio (ER vs CMR)	Regulation
CA03g35150	Polygalacturonase	0.417	Down
CA12g08070	Peptidyl-prolyl cis–trans isomerase	0.79	Down
CA06g26470	Peptidyl-prolyl cis–trans isomerase	1.457	Up
CA02g23570	Leucine-rich repeat protein	0.695	Down
CA05g14990	Glutathione S-transferase	0.763	Down
CA10g14270	Glutathione S-transferase	1.809	Up
CA09g03120	Glutathione S-transferase	1.564	Up
CA06g02950	Glutathione S-transferase	1.332	Up
CA07g06720	Cysteine protease	0.403	Down
CA12g16570	Cysteine protease	0.529	Down
CA03g15860	Pectin methlyesterase	0.707	Down
CA03g36980	Pectin methylesterase	1.203	Up
CA10g05650	Disease resistance response protein	0.393	Down
CA02g15780	Polyphenol oxidase	0.511	Down
CA01g31060	Pathogenesis-related protein	0.552	Down
CA00g28760	Pathogenesis-related protein	0.792	Down
CA03g04260	Pathogenesis-related protein	0.476	Down
CA01g03050	Pathogenesis-related protein	0.387	Down
CA08g09990	Beta-1,3-glucanase	0.826	Down
CA01g12140	Beta-1,3-glucanase	0.437	Down
CA05g12260	Beta-1,3-glucanase	0.237	Down
CA01g09600	Beta-1,3-glucanase	0.541	Down
CA05g12210	Beta-1,3-glucanase	0.336	Down
CA10g13810	Beta-1,3-glucanase	0.244	Down
CA01g11410	Peroxidase-like	0.414	Down
CA07g12090	Peroxidase-like	0.209	Down
CA04g14020	Peroxidase-like	0.386	Down
CA02g22040	Peroxidase-like	0.647	Down
CA08g15620	Peroxidase-like	0.381	Down
CA04g22110	Peroxidase-like	0.658	Down
CA02g30850	Peroxidase-like	0.646	Down
CA09g07310	Peroxidase-like	0.475	Down
CA11g02470	Late blight resistance protein	1.239	Up
CA04g13070	Pathogen-related protein-like	2.368	Up
CA08g07290	Aquaporins	1.413	Up
CA10g15710	Aquaporins	1.543	Up
CA09g02770	Aquaporins	1.27	Up
CA09g03700	Ascorbate peroxidase	1.263	Up
CA06g06260	Ascorbate peroxidase	1.266	Up

In the comparative proteome analysis of E_EL and CM_EL, we identified several DEPs associated with *P. capsici* infection, as detailed in Table 2. Notably, one leucine-rich repeat protein CA11g10920, one disease resistance response protein CA08g18950, one polygalacturonase CA03g35150, one β-1,3-glucanase CA05g12260, one disease resistance protein CA00g73770, one expansin-like protein CA00g52560, one thioredoxin peroxidase CA01g23340, two nitrite reductase, three peptidyl-prolyl cis–trans isomerases, and four peroxidase-like proteins were identified, all of which were downregulated in CM_EL. Conversely, one late blight resistance protein CA11g02470, one pectin methylesterase CA03g36980, one calcium-sensing receptor protein CA03g29260, one calcineurin B-like protein CA03g14850, one pectinesterase CA06g00420, and three glutamate S-transfer proteins exhibited upregulation in CM_EL. Furthermore, we identified three polyphenol oxidases, three aquaporins, and three pathogenesis-related proteins that exhibited differential expression between E_EL and CM_EL.

**Table 2:** Identification of *Phytophthora capsici* resistance-related differentially expressed proteins between E_EL and CM_EL.

DEPs_ID	Annotation	Mean_Ratio (E_EL vs CM_EL)	Regulation
CA11g10920	Leucine-rich repeat protein	0.768	Down
CA08g18950	Disease resistance response protein	0.622	Down
CA03g35150	Polygalacturonase	0.636	Down
CA05g12260	Beta-1,3-glucanase	0.717	Down
CA10g07200	Nitrite reductase	0.78	Down
CA00g41210	Nitrite reductase	0.808	Down
CA01g28400	Peptidyl-prolyl cis–trans isomerase	0.631	Down
CA00g19810	Peptidyl-prolyl cis–trans isomerase	0.725	Down
CA03g33980	Peptidyl-prolyl cis–trans isomerase	0.813	Down
CA02g26770	Polyphenol oxidase	1.806	Up
CA02g15780	Polyphenol oxidase	0.658	Down
CA01g33890	Polyphenol oxidase	0.722	Down
CA00g73770	Disease resistance protein	0.827	Down
CA00g52560	Expansin-like protein	0.79	Down
CA10g15710	Aquaporins	0.478	Down
CA11g19000	Aquaporins	0.716	Down
CA03g34630	Aquaporins	1.268	Up
CA01g11410	Peroxidase-like	0.753	Down
CA02g30850	Peroxidase-like	0.571	Down
CA09g07330	Peroxidase-like	0.666	Down
CA12g03520	Peroxidase-like	0.682	Down
CA01g23340	Thioredoxin peroxidase-like	0.823	Down
CA11g02470	Late blight resistance protein	1.342	Up
CA03g36980	Pectin methylesterase	1.624	Up
CA03g29260	Calcium sensing receptor	1.291	Up
CA03g14850	Calcineurin B-like protein	1.508	Up
CA01g31060	Pathogenesis-related protein	0.615	Down
CA12g13830	Pathogenesis-related protein	1.258	Up
CA00g45250	Pathogenesis-related protein	1.859	Up
CA06g00420	Pectinesterase	1.484	Up
CA05g19020	Glutathione S-transferase	1.217	Up
CA07g16210	Glutathione S-transferase	1.355	Up
CA07g16200	Glutathione S-transferase	1.719	Up

Furthermore, seven DEPs related to *P. capsici* were identified based on the functional analysis of DEPs in ER versus CMR and E_EL versus CM_EL, and their expression trends were consistent between the roots and leaves of ‘ZCM334’ grafted and ‘Early Calwonder’ self-rooted grafted plants. These DEPs included six downregulated proteins (CA03g35150, CA01g11410, CA05g12260, CA02g30850, CA02g15780, and CA01g31060) and one upregulated protein (CA03g36980) in both the roots and leaves of ‘ZCM334’ grafted plants. This suggests that grafting may affect the expression of *P. capsici*-related proteins, thereby affecting pepper resistance to *P. capsici*. Identification of these DEPs provides valuable insights for further exploration of disease resistance mechanisms in grafted pepper.

### Functional classification of DEPs

**Figure 4:**
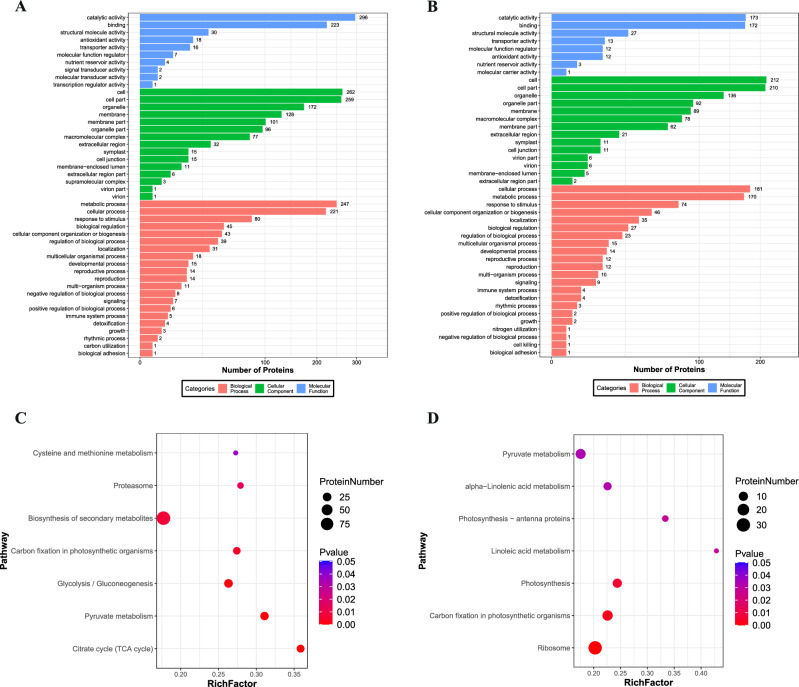
GO classification and KEGG pathway assignment of differentially expressed proteins in the roots and leaves of ‘ZCM334’ grafted plants and ‘Early Calwonder’ self-rooted grafted plants. (**A**) GO classification of differentially expressed proteins between ER and CMR. (**B**) GO classification of differentially expressed proteins between E_EL and CM_EL. (**C**) KEGG pathway assignment of differentially expressed proteins between ER and CMR. (**D**) KEGG pathway assignment of differentially expressed proteins between E_EL and CM_EL. ER, roots of ‘Early Calwonder’ self-rooted grafted plants; CMR, roots of ‘ZCM334’ grafted plants; E_EL, leaves of ‘Early Calwonder’ self-rooted grafted plants; CM_EL, leaves of ‘ZCM334’ grafted plants.

To explore the functions of the DEPs, we conducted Gene Ontology (GO) and Kyoto Encyclopedia of Genes and Genome (KEGG) enrichment analyses of the DEPs in the ER versus CMR and E_EL versus CM_EL groups (Tables S2 and S3). These DEPs were annotated in 49 GO terms, with the majority falling under “metabolic process,” “cellular process,” and “response to stimulus” in the biological process. They were also present in the “cell,” “cell part,” “membrane,” and “organelle” categories under cellular components and in the “catalytic activity,” “binding,” and “structural molecule activity” categories under molecular function (Fig. 4A,B). According to the KEGG enrichment analysis, the DEPs between ER and CMR were enriched in 18 pathways, including seven significantly enriched KEGG metabolic pathways (*p* ≤ 0.05), most of which were significantly enriched in “biosynthesis of secondary metabolites,” “pyruvate metabolism,” “glycolysis/gluconeogenesis,” “carbon fixation in photosynthetic organisms,” and “citrate cycle (TCA cycle) metabolic” pathways (Fig. 4C). Most DEPs between E_EL and CM_EL were significantly enriched in “ribosome,” “carbon fixation in photosynthetic organisms,” “photosynthesis,” “pyruvate metabolism,” and “alpha-linolenic acid metabolism metabolic” pathways (Fig. 4D).

### Transcriptional expression analysis of DEPs

**Figure 5:**
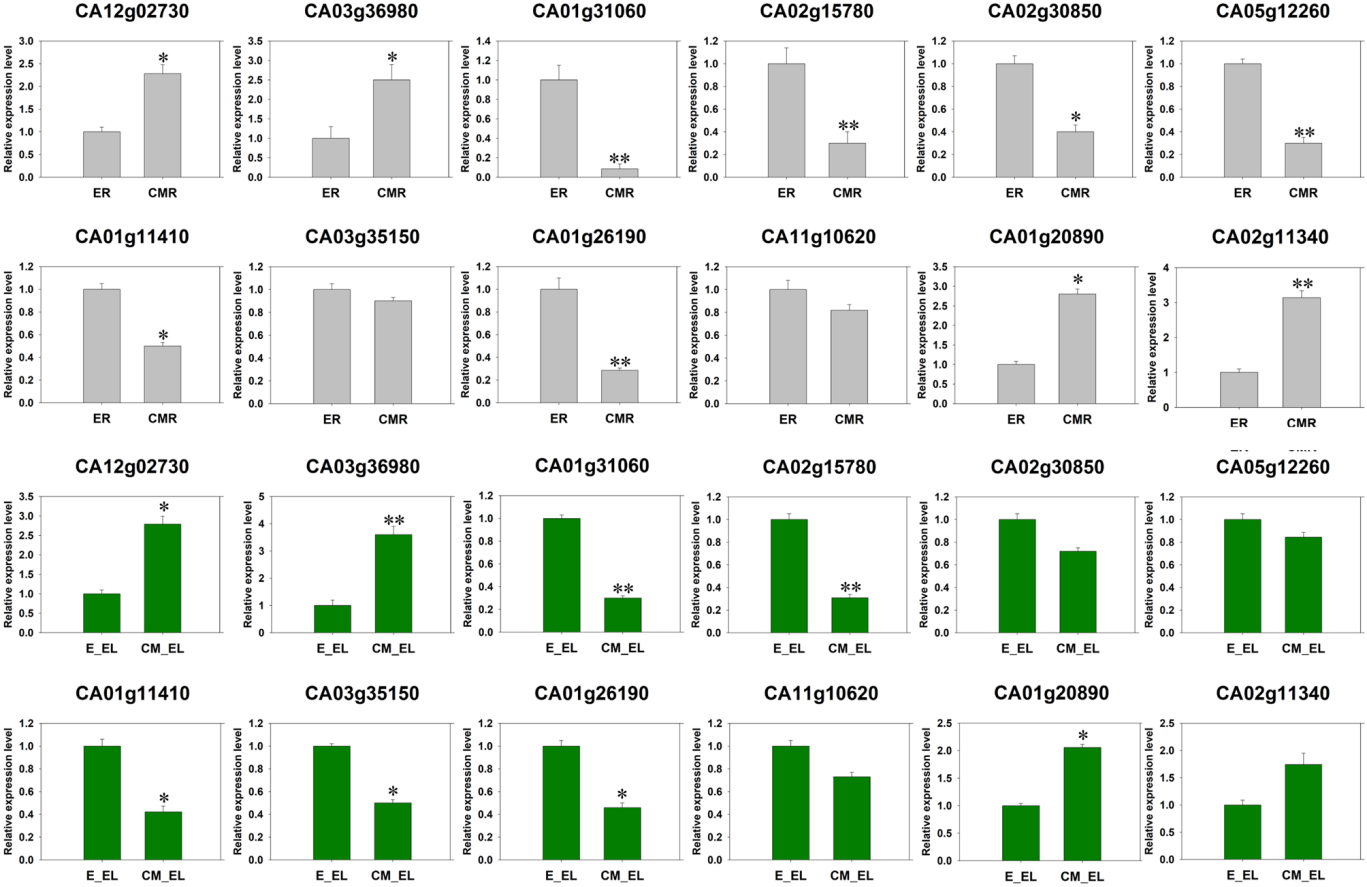
qRT-PCR-based quantification of 12 differentially expressed proteins in ER versus CMR and E_EL versus CM_EL at 12 h post-inoculation with *Phytophthora capsici*. ER, roots of ‘Early Calwonder’ self-rooted grafted plants; CMR, roots of ‘ZCM334’ grafted plants; E_EL, leaves of ‘Early Calwonder’ self-rooted grafted plants; CM_EL, leaves of ‘ZCM334’ grafted plants. **P < 0.01 and *P < 0.05, based on Duncan’s test.

To validate the iTRAQ data, we selected 12 DEPs for qRT-PCR analysis in the roots and leaves of ‘ZCM334’ grafted and ‘Early Calwonder’ self-rooted grafted plants 12 h after inoculation with *P. capsici*. These DEPs included seven DEPs related to *P. capsici* and five DEPs with unknown functions. qRT-PCR results showed that the genes encoding pectin methylesterase CA03g36980 and three proteins with unknown function were upregulated in the roots and leaves of ‘ZCM334’ grafted plants. Conversely, the genes encoding pathogenesis-related protein CA01g31060, polyphenol oxidase CA02g15780, peroxidase-like proteins CA02g30850 and CA01g11410, beta-1,3-glucanase CA05g12260, polygalacturonase-like protein CA03g35150, and two proteins with unknown function were downregulated in the roots and leaves of ‘ZCM334’ grafted plants. The gene expression trends for all 12 DEPs identified using qRT-PCR were consistent with those determined using iTRAQ, indicating that the iTRAQ results were reliable (Fig. 5).

**Figure 6:**
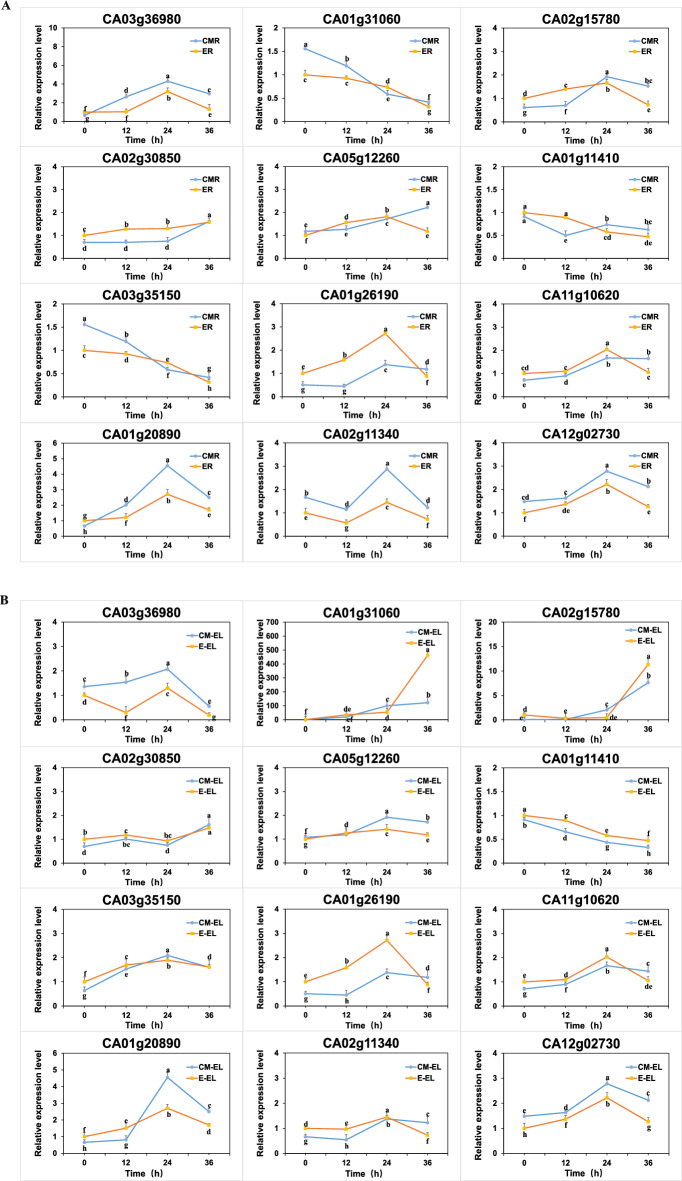
qRT-PCR-based quantification of 12 differentially expressed proteins in ER versus CMR (**A**) and E_EL versus CM_EL (**B**) at different stages post-inoculation with *Phytophthora capsici.* ER, roots of ‘Early Calwonder’ self-rooted grafted plants; CMR, roots of ‘ZCM334’ grafted plants; E_EL, leaves of ‘Early Calwonder’ self-rooted grafted plants; CM_EL, leaves of ‘ZCM334’ grafted plants. Different letters indicate significant differences among the different treatments according to the least significant difference (LSD) test at *P* < 0.05.

The uniform expression trends observed in both the leaves and roots of ‘ZCM334’ grafted and ‘Early Calwonder’ self-rooted grafted plants for these 12 genes, along with the known association of seven of them with *P. capsici*, suggest that these genes likely play a role in enhancing resistance to *Phytophthora* blight through grafting. In other words, the differential expression of these genes in ‘ZCM334’ grafted plants may enhance plant disease resistance. To further explore the function of these 12 DEPs, we analyzed their gene expression patterns in the roots and leaves of ‘ZCM334’ grafted and ‘Early Calwonder’ self-rooted grafted plants at different stages (0 h, 12 h, 24 h, and 36 h) after inoculation with *P. capsici* (Fig. 6)*.* Among these genes, CA03g36980 and five unknown functional proteins exhibited the highest expression in the roots and leaves of ‘ZCM334’ grafted and ‘Early Calwonder’ self-rooted grafted plants 12 h after *P. capsici* inoculation*.* With the prolongation of inoculation time with *P. capsici*, the expression of CA01g31060 and CA03g35150 in the ER and CMR gradually decreased, while that of CA01g31060 in E_EL and CM_EL gradually increased, reaching the highest level 36 h post-inoculation, while the expression of CA03g35150 in the leaves peaked 12 h post-inoculation before declining. After inoculation with *P. capsici*, the expression of CA01g11410 considerably decreased in both the leaves and roots of ‘ZCM334’ grafted and ‘Early Calwonder’ self-rooted grafted plants. In the ER and CMR, CA02g15780 showed the highest expression 12 h after *P. capsici* inoculation, whereas in E_EL and CM_EL, CA02g15780 showed the highest expression after 36 h of *P. capsici* inoculation. The expression of CA02g30850 was the highest in the roots and leaves of ‘ZCM334’ grafted and ‘Early Calwonder’ self-rooted grafted plants 36 h after *P. capsici* inoculation, but the expression of CA02g30850 in the roots continued to increase; contrastingly, the expression in the leaves first increased, then decreased, and then increased. In E_EL and CM_EL, the expression of CA05g12260 was highest 24 h after *P. capsici* inoculation, but in the roots of ‘ZCM334’ grafted plants, the expression of CA05g12260 increased as the inoculation time extended. In the roots of ‘Early Calwonder’ self-rooted grafted plants, the expression of CA05g12260 first increased and then decreased; the expression was highest after 24 h of *P. capsici* inoculation.

## Discussion

*Phytophthora* blight, caused by *P. capsici* Leonian, is a highly destructive soil-borne disease posing a significant threat to pepper cultivation across various countries and regions worldwide^23–25^. The pathogenic spores of *Phytophthora* blight can survive in the soil for a long time, and an environment suitable for the growth of pepper is also suitable for the growth and reproduction of *P. capsici*, leading to frequent outbreaks of *Phytophthora* blight^26^. *Phytophthora* blight encompasses diverse manifestations, a multitude of physiological races, and a high susceptibility to mutation within the pathogen^27^. Moreover, the genetic law of pepper resistance to *P. capsici* is complex, and the mechanism of resistance is unclear, hampering progress in resistance breeding efforts.

Grafting, an important cultivation measure for preventing soil-borne diseases and overcoming continuous cropping obstacles, is widely used in vegetable production. It can improve the resistance of vegetable crops to diseases, drought, salt, and other stresses, leading to increased yields and improved quality. Grafting with highly resistant or immune rootstocks can prevent or control soil-borne diseases^28,29^. Grafted rootstocks maintain the genetic makeup of the scion while significantly enhancing scion resistance. These disparities inevitably encompass various transcriptional and translational variations. Therefore, the efficient and accurate identification of DEPs in scions grafted onto different rootstocks, despite having the same susceptible scion, is pivotal for comprehending how grafting improves pepper's disease resistance.

The pepper strain ‘ZCM334’, which is completely immune to *P. capsici* physiological race 3, was obtained through multiple generations of self-breeding following inoculation identification. Inoculating ‘ZCM334’ with *P. capsici* race 3 resulted in normal plant growth without any signs of infection. Subsequently, we conducted grafting experiments using the ‘ZCM334’ immune strain as rootstocks and the susceptible ‘Early Calwonder’ strain as scions. Using the root inoculation method, an inoculation experiment was conducted on plants grafted onto different rootstocks using the same susceptible scions. Phenotypic observation showed that the grafted plants of ‘ZCM334’ had strong resistance to *P. capsici*, and the leaves began to darken at 24 h post-inoculation. This delayed onset of the disease marked contrast to ‘Early Calwonder’ self-rooted grafted plants, where new leaves at the growth point began darkening at 12 h post-inoculation, resulting in a quicker disease incidence. In these plants, the top stem turned black, and the leaves rapidly withered. These differences in disease resistance suggest that disparities in the rootstocks may lead to the differential expression of specific genes and proteins, ultimately affecting resistance among plants grafted with the same susceptible scion on different rootstocks.

This study used iTRAQ technology to sequence the leaves and roots of ‘ZCM334’ grafted and ‘Early Calwonder’ self-rooted grafted plants. In total, 478 DEPs were identified between ER and CMR, and 349 DEPs were identified between E_EL and CM_EL. From these DEPs, we selected certain DEPs that play important roles in the infection process of *P. capsici*, laying the foundation for exploring the molecular mechanism underlying pepper’s resistance to *P. capsici*. *CALRR1* encodes a leucine-rich repeat (LRR) protein that is strongly induced in pepper leaves after infection with *P. capsici*, *Colletotrichum gloeosporioides,* and *Colletotrichum coccodes*. Inoculation of living bacterial cells and treatment with dead cells of pathogenic or non-pathogenic bacteria trigger the accumulation of *CALRR1* transcripts. *CALRR1* may play a role in protecting phloem cells from biotic and abiotic stresses affecting phloem function^30^. In this study, we discovered two LRR proteins; CA02g23570 was differentially expressed between ER and CMR, and CA11g10920 was differentially expressed between E_EL and CM_EL. Both of these proteins were downregulated in ‘ZCM334’ grafted plants, which may be closely related to resistance to *P. capsici.* In plants, *Phytophthora* secretes numerous effectors that manipulate host processes to create an environment conducive to pathogen colonization. Fan et al. reported that *P. capsici* RXLR effector and PcAvr3a12, members of the Avr3a effector family, inhibit plant immunity by targeting and inhibiting host plant peptidyl-prolyl cis–trans isomerase (PPIase), which is required for endoplasmic reticulum stress-mediated plant immunity^31^. CA01g28400, CA00g19810, and CA03g33980 were downregulated in CM_EL compared to those in E_EL. In addition, two differentially expressed PPIases (CA12g08070 and CA06g26470) were identified between ER and CMR. These DEPs may interact with RXLR and Avr3a effectors to regulate plant immunity following *P. capsici* infection*.*

Caamal-Chan et al. demonstrated that nitrate reductase activity may be related to specific host defense responses^32^. *P. capsici* and plant defense hormones can also alter nitrate reductase enzyme activity. Nitrite reductases CA10g07200 and CA00g41210 were downregulated in CM_EL compared with those in E_EL, which may be related to the defense response of *P. capsici* after leaf infection. Infection with *P. capsici* or *Colletotricum coccodes* induces the expression of three types of peroxidases in pepper tissues: peroxidase-like (CAPO1), thioredoxin peroxidase-like (CAPOT1), and ascorbate peroxidase (CAPOA1). During programmed cell death, significant accumulation of H_2_O_2_ and decreases in peroxidase activity may result from the strong inhibition of CAPOA1, CAPOT1, and CAPO1 gene expression^33^. Our iTRAQ data pinpointed eight CAPO1 proteins that were differentially expressed between ER and CMR, along with four CAPO1 proteins and one CAPOT1 protein that were differentially expressed between E_EL and CM_EL. These DEPs were all downregulated in the roots or leaves of ‘ZCM334’ grafted plants, indicating that when infected by *P. capsici*, a decrease in the expression of these proteins may lead to a decrease in peroxidase activity, resulting in rapid plant disease onset and wilting.

In *Nicotiana benthamiana*, the overexpression of a calcium-sensing receptor significantly enhances the resistance of plants to *P. capsici* and *Sclerotinia sclerotiorum*, indicating that the calcium-sensing receptor is a promising disease resistance breeding gene^34^. Calcineurin B-like proteins (CBLs) are major calcium ion sensors that interact with CBL-interacting protein kinases (CIPKs) to regulate plant growth and development^35^. The CBL–CIPK regulatory network participates in stress responses to *P. capsici*, salicylic acid, abscisic acid, ethylene, heat stress, and cold stress^36^. In this study, the calcium-sensing receptor protein CA03g29260 and calcineurin B-like protein CA03g14850 were identified, both of which were upregulated in CM_EL compared with those in E_EL. This may be closely related to the resistance of plant leaves to *P. capsici* infection. An expansin-like protein, PcEXLX1, has been identified in the apoplastic fluid of *Nicotiana benthamiana* infected with *P. capsici*. Knockdown mutants of PcEXLX1 exhibited increased virulence, while overexpression reduced virulence. In addition, the prokaryotic expression of PcEXLX1 activated various plant immune responses. Moreover, overexpression of *PcEXLX1* homolog in *Nicotiana benthamiana* increased plant resistance to *P. capsici*^37^. In this study, CA00g52560 was identified as an expansin-like protein downregulated in CM_EL compared with that in E_EL. This protein may act as an effector of plant immune induction activity, thereby participating in the resistance to *P. capsici* infection in plant leaves. Extracellular inducer proteins of *Phytophthora* species can induce specific defense responses in Crossaceae and Solanaceae. Bacteria express the inducer gene as a fusion protein containing glutathione S-transferase, which produces a bioactive protein capable of inducing hypersensitivity in tobacco^38^. Four glutathione S-transferases were identified between ER and CMR, three of which were upregulated in the CMR, and three glutathione S-transferases were identified between E_EL and CM_EL, all of which were upregulated in CM_EL. These DEPs may induce defense responses in pepper plants after *P. capsici* infestation. Cysteine proteases are involved in plant development and stress responses^39^. Xiao et al. reported that a new cysteine protease gene, *CaCP*, is related to senescence in pepper leaves^40^. High salinity, *P. capsici*, mannitol, and other abiotic and biological stressors can also significantly induce the expression of *CaCP*. Two cysteine protease proteins, CA07g06720 and CA12g16570, were screened between ER and CMR, and both were upregulated in CMR. These findings may be linked to differences in the defense response of the roots of ‘ZCM334’ grafted and ‘Early Calwonder’ self-rooted grafted plants to *P. capsici.*

In the proteomic sequencing data of this study, we selected seven DEPs related to *P. capsici* that exhibited consistent expression patterns in both the roots and leaves of ‘ZCM334’ grafted and ‘Early Calwonder’ self-rooted grafted plants. Among these DEPs, one was upregulated, while six were downregulated in the roots and leaves of ‘ZCM334’ grafted plants. We further analyzed their expression patterns using qRT-PCR. In plants, the cell wall plays an important role in resisting pathogen attack. Polygalacturonases (PGs) are cell wall-degrading enzymes that play important roles in straminophilic pathogens^41^. To understand the relationship between PGs and *P. capsici*, Sun et al. identified *Pcipg2* from the genomic library of a highly virulent *P. capsici* strain that was strongly expressed in pepper leaves after inoculation with *P. capsici*^42^. In our study, we observed a significant downregulation of polygalacturonase CA03g35150 in the roots and leaves of ‘ZCM334’ grafted plants. This may imply an essential role of CA03g35150 in cell wall development, leading to variations in leaf characteristics between ‘ZCM334’ grafted and ‘Early Calwonder’ self-rooted grafted plants. The qRT-PCR results showed that in ER and CMR, with the invasion of *P. capsici*, the expression of CA03g35150 significantly decreased, indicating that the plant may reduce cell wall degradation and achieve defensive and protective effects by inhibiting the expression of CA03g35150. In one study on the resistance to root and base rot disease caused by *P. capsici*, the polyphenol oxidase activity in the resistant materials was significantly increased, indicating a significant correlation between resistance to *P. capsici* and the level of polyphenol oxidase activity^43^. CA02g15780 is a protein encoding polyphenol oxidase that is significantly downregulated in the roots and leaves of ‘ZCM334’ grafted plants. After inoculation with *P. capsici* on the leaves of ‘ZCM334’ grafted and ‘Early Calwonder’ self-rooted grafted plants, the expression of CA02g15780 gradually increased, reaching its highest level 36 h post-inoculation. This indicates that in the later stage of inoculation with *P. capsici*, plants can resist the infection of pathogenic bacteria by promoting the high expression of CA02g15780.

*P.* *capsici* secretes various pectin methylesterases (PMEs) at various stages of infection, causing damage to many crops^44^. Feng et al. identified a PME gene, *pcpme6*, from the genomic library of the highly virulent *P. capsici* strain SD33, and its expression gradually increased after *P. capsici* infected pepper leaves^45^. In our iTRAQ data, we identified a PME protein, CA03g36980, with significantly higher expression in the roots and leaves of ‘ZCM334’ grafted plants after *P. capsici* infection than in those of ‘Early Calwonder’ self-rooted grafted plants. The highest expression of CA03g36980 was observed in the leaves and roots of ‘ZCM334’ grafted plants and ‘Early Calwonder’ self-rooted grafted plants at 24 h post-inoculation with *P. capsici*, and the expression in CMR and CM_EL was higher than that in ER and E_EL. This indicates that CA03g36980 may be induced after inoculation, contributing to plant resistance against pathogen infection. Plants employ a wide range of mechanisms to combat various biotic and abiotic stresses, including the accumulation of pathogenesis-related (PR) proteins. PR proteins play a crucial role in plant defense responses against pathogens. For example, the *PnPR-1* gene plays an important role in regulating the first layer of plant defense against *P. capsici* infection^46^. In this study, we identified a PR protein, CA01g31060, which was downregulated in the roots and leaves of ‘ZCM334’ grafted plants. Some studies have reported that genes encoding PR proteins, such as β-1,3-glucanase and chitinase, are induced in the leaves and stems of pepper plants by *P. capsici*^32,46^. Since the expression of PR genes in incompatible interactions is higher than that in compatible interactions, the time and intensity of gene expression may lead to differences in sensitivity and resistance^32^. β-1,3-glucanase plays an important role in the response to *P. capsici*, which may be directly related to high-level resistance^47^. CA05g12260, encoding β-1,3-glucanase, was also identified in our study. At the 36th hour post-inoculation with *P. capsici*, the expression of CA05g12260 in the roots and leaves of ‘ZCM334’ grafted plants was significantly higher than that of ‘Early Calwonder’ self-rooted grafted plants. This indicates that CA05g12260 may play an important role in the resistance process of pepper to *P. capsici*. Furthermore, five proteins with unknown functions all reached their highest expression levels at 24 h post-inoculation with *P. capsici*, indicating that this may be an important stage for the gene response to pathogen infection.

In conclusion, this study identified DEPs between the roots and leaves of ‘ZCM334’ grafted and ‘Early Calwonder’ self-rooted grafted plants inoculated with *P. capsici* through proteomic analysis. These DEPs were significantly enriched in the biosynthesis of secondary metabolites, carbon fixation in photosynthetic organizations, and pyruvate metabolism pathways. Some defense genes that play important roles in pepper resistance to *P. capsici* infection were screened from DEPs in the roots and leaves. Among them, 12 DEPs, including pectin methylesterase, pathogenesis-related protein, polyphenol oxidase, peroxidase-like proteins, beta-1,3-glucanase, polygalacturonase-like proteins, and five proteins with unknown functions, showed consistent expression trends in the leaves and roots. However, it remains unclear how these DEPs regulate the resistance of grafted plants to *P. capsici*. In the future, in-depth functional validation should be conducted on these selected DEPs. Our research findings contribute to a deeper understanding of the response of pepper to *P. capsici* infection and the molecular mechanisms through which grafting enhances pepper resistance to *Phytophthora* blight. Additionally, this study identifies potential candidate genes for further research into the regulatory network of pepper resistance to *P. capsici*.

## Materials and methods

### Plant materials, pathogen inoculation, and phenotype identification

In the initial phases of our study, we identified the ‘CM334’ material, known for its high resistance to *P. capsici*, sourced from the Asian Vegetable World Vegetable Center. After multiple generations of self-pollination, we obtained a highly inbred line called ‘ZCM334,’ which demonstrated complete immunity to *Phytophthora* blight. In this study, we utilized the immune ‘ZCM334’ strain and the susceptible ‘Early Calwonder’ strain, employing ‘ZCM334’ as rootstocks and ‘Early Calwonder’ as scions. The plants were planted in a solar greenhouse at the Liaoning Academy of Agricultural Sciences. The split grafting method was used once the rootstock seedlings had grown to 5–6 leaves and the scion seedlings had reached four leaves. The upper part of the rootstock was cut off, leaving the first pair of true leaves, and the stem was separated from the middle by a 1 cm gap. Similarly, the lower part of the scion was cut, retaining three to four true leaves. The stem of the scion was then crafted into a 1 cm wedge and inserted into the notch of the rootstock. After 20 d, ‘ZCM334’ grafted plants and ‘Early Calwonder’ self-rooted grafted plants were subjected to inoculation, followed by phenotypic identification and sampling.

For this study, we utilized the *P. capsici* physiological race 3, referred to as ‘ZY14,’ which was provided by Liu Changyuan, a researcher in the Horticultural Disease Research Office of the Plant Protection Institute, Liaoning Academy of Agricultural Sciences. Initially, ‘ZY14’ underwent rejuvenation and propagation, followed by transfer to V8 medium and cultivation at 28 °C for 7 d. Once the mycelium fully colonized the culture dish, we cut the culture medium into 2 cm^3^ pieces and soaked them in sterile water for 3 d. After sporulation, the spores were placed in a 10 °C incubator for 1 h to promote the release of spores. After incubating for 30 min at 24 °C, the spore concentration was calculated using the blood cell counting board. The spore concentration of *P. capsici* was then diluted with sterile water to a concentration of 2000 spores/mL. Subsequently, we transferred both the ‘ZCM334’ grafted plants and the ‘Early Calwonder’ self-rooted grafted plants to an artificial climate room. We evenly and comprehensively sprayed the pepper leaves with spores, covered them with plastic film post-inoculation, and maintained a temperature of 25–30 °C and humidity at 90%.

Observations of ‘ZCM334’ grafted plants and ‘Early Calwonder’ self-rooted grafted plants were conducted at 0, 12, 18, 24, 36, and 48 h following inoculation. Specifically, we focused on the second new leaves, harvested from the growth point at these time intervals. Paraffin section analysis was performed on longitudinal sections of the leaves, following the procedures detailed in Zhou et al.^48^. Additionally, we collected samples from the leaves and roots of ‘ZCM334’ grafted plants and the ‘Early Calwonder’ self-rooted grafted plants at 0, 12, 24, and 36 h after *P. capsici* inoculation. These samples were frozen in liquid nitrogen and stored at − 80 °C in a refrigerator for subsequent RNA extraction and qRT-PCR analysis. The leaf samples represented a composite of three plants, while the root samples were a mixture of main and fibrous roots collected from three plants. For proteomic analysis, leaf and root samples of ‘ZCM334’ grafted plants and ‘Early Calwonder’ self-rooted grafted plants were gathered at 12 h post-inoculation.

### Protein preparation

The root and leaf samples of ‘ZCM334’ grafted plants and ‘Early Calwonder’ self-rooted grafted plants were ground into powder in liquid nitrogen. An appropriate amount of protein lysate was added, followed by the addition of 1 mM PMSF and 2 mM EDTA. After 5 min, 10 mM DTT was introduced. Subsequently, we subjected the mixed solution to 15 min of ultrasonic treatment and centrifuged it at 25,000 × *g* for 20 min. The supernatant was collected, and we added five times the volume of acetone, allowing it to sit at − 20 °C for 2 h. After another centrifugation step at 16,000 × *g* for 20 min, we introduced protein lysate to the sediment and added 1 mM PMSF and 2 mM EDTA. After a 5-min incubation, 10 mM DTT was added. Following 15 min of ultrasonic treatment, the mixed solution was centrifuged at 25,000 × *g* for 20 min, and 10 mM DTT was introduced to the supernatant for 1 h. Subsequently, we included 55 mM IAM for a 45-min dark incubation. We added acetone and placed it at − 20 °C for 2 h. After a final centrifugation step at 25,000 × *g* for 20 min, we introduced 200 μL of 0.5 M TEAB to the precipitate, followed by 15 min of ultrasonication. Another centrifugation at 25,000 × *g* for 20 min was performed, and we quantified the protein using the Bradford method^49^. The protein quality of the roots and leaves of grafted and self-rooted pepper plants was determined using sodium dodecyl sulfate–polyacrylamide gel electrophoresis. The protein samples were then stored at − 80 °C for further experiments.

### Protein enzymolysis, iTRAQ labeling, and SCX fractionation

We accurately extracted a total of 100 μg protein from each sample. Trypsin was added at a protein-to-enzyme ratio of 20:1, and enzymatic digestion occurred at 37 °C for 4 h. Afterward, trypsin was added again following the original ratio, and the digestion continued at 37 °C for 8 h. The resulting peptide segments were dried using a vacuum centrifugal pump. For iTRAQ labeling, we redissolved the peptide segments in 0.5 M TEAB and used an 8-plex iTRAQ reagent (Applied Biosystems, Foster, CA, USA). Each peptide group was labeled with different iTRAQ tags. The sample marker information is as follows: roots of ‘ZCM334’ grafted plants (CMR):117, roots of ‘Early Calwonder’ self-rooted grafted plants (ER):118, leaves of ‘Early Calwonder’ self-rooted grafted plants (E_EL):119, and leaves of ‘ZCM334’ grafted plants (CM_EL):121. After incubating the labeled peptide segments at 25 °C for 2 h, the peptide segments of each group were mixed for subsequent liquid phase separation.

Liquid phase separation of the samples was performed using an LC-20AB liquid phase system (Shimadzu, Kyoto, Japan) and Ultremex SCX separation columns (4.6 × 250 mm). The labeled mixed peptides were redissolved in 4 mL buffer A (25 mM NaH_2_PO_4_ in 25% ACN, pH 2.7). Within the separation column, gradient elution was performed at a rate of 1 mL/min. This involved elution in 5% buffer B (25 mM NaH_2_PO_4_, 1 M KCl in 25% ACN, pH 2.7) for 7 min, followed by a 20 min linear gradient to increase buffer B from 5 to 60%. Finally, the proportion of buffer B was increased to 100% within 2 min and maintained for 1 min before returning to 5% equilibrium for 10 min. The whole elution process was monitored at 214 nm absorbance, and 12 components were selected. Each fraction was desalted separately using a Strata X desalting column and frozen.

### LC–ESI–MS/MS analysis based on Triple TOF 5600

We dissolved each desalted component in buffer A (5% ACN, 0.1% FA) to a concentration of approximately 0.5 μg/μL. After centrifuging at 20,000 × *g* for 10 min and removing insoluble substances, we loaded 5 μL of each component for separation using a Shimadzu LC-20AD nanoliter liquid chromatograph. The columns used included a trap column and an analysis column. The separation procedure was as follows: the sample was loaded onto the trap column at a flow rate of 8 μL/min for 4 min. This was followed by an analysis gradient at a total flow rate of 300 nL/min to introduce the sample into the analysis column, where it was separated and transmitted to the mass spectrometry system. We eluted at 5% buffer B (95% ACN, 0.1% FA) for 5 min, followed by a 35 min linear gradient to increase buffer B from 5 to 35%. Subsequently, buffer B was increased to 60% within 5 min, further increased to 80% within 2 min, maintained for 2 min, and finally returned to 5% within 1 min, balancing under this condition for 10 min. A Triple TOF 5600 system (AB SCIEX, Concord, Ontario, Canada) was used for data acquisition. Refer to Zhou et al. for the parameter settings of the machine^50^. The ion source was a Nanospray III (AB SCIEX, Concord, ON), and a quartz needle (New Objectives, Woburn, MA, USA) served as the emitter. The ion fragmentation energy was set at 35 ± 5 eV^50^. To prevent repeated fragmentation of the same parent ion within half of the peak time (approximately 15 s), dynamic exclusion of the parent ion was applied.

### Protein data analysis and functional annotation

We utilized Proteome Discoverer 1.2 software to convert the original data files obtained from Orbitrap to MGF files. Protein identification was performed using the Mascot search engine (Matrix Science, London, UK; version 2.3.02) against Pepper.v.1.55. PEP database (http://peppergenome.snu.ac.kr/download.php; 34,598 sequences). The fragment mass tolerance and peptide mass tolerance were set to a maximum of 0.1 Da and 0.05 Da, respectively. The maximum number of missed cleavages was one. The variable modifications were Gln- > pyro-Glu (N-term Q), oxidation (M), and deamidation (NQ), while the fixed modifications were carbamidomethyl (C), iTRAQ8plex (N-term), and iTRAQ8plex (K). The peptide charges were set to + 2 and + 3. By selecting the decoy checkbox, an automatic decoy database search using Mascot generated a random sequence of the database to validate results. Only peptides with a 95% confidence interval greater than identity were considered identified. For quantification, proteins needed to be identified with at least two unique spectra. Simultaneously, a peptide spectrum containing all six reported ions was used for quantification. Quantitative protein ratios were weighted and standardized using the median ratio in Mascot. Proteins were considered differentially expressed when the abundance ratio exceeded 1.2 times, with a *p*-value < 0.05, confirmed through statistical analysis.

To annotate gene functions, we employed GO enrichment analysis based on the annotation of known genes (proteins). GO classifies gene functions into “biological processes,” “molecular functions,” and “cellular components.” The Blast2GO program was used for protein function annotation (http://www.geneontology.org/). The KEGG database was used to annotate the information identified in the biological pathways. Through significant pathway enrichment, the main biochemical metabolic pathways and signal transduction pathways involved in the DEPs were identified^51^. BLASTX/BLASTP 2.2.24+ was used to map the DEPs to the pathways identified using the KEGG database (http://www.genome.jp/kegg/genes.html).

### qRT-PCR verification

To verify the reliability of proteomic sequencing and the expression patterns of DEPs related to the resistance of *P. capsici*, 12 DEPs were selected for qRT-PCR analysis in the CMR, ER, E_EL, and CM_EL samples at 0, 12, 24, and 36 h after inoculation with *P. capsici*. Total RNA from the CMR, ER, E_EL, and CM_EL samples was extracted using a TRIzol kit (Invitrogen, USA). Oligo-dT magnetic beads were used to purify mRNA from total RNA, and SuperScript III was used to synthesize cDNA according to the manufacturer's instructions. The cDNA was diluted (1:10) for qRT-PCR, and primers were designed using Primer 5.0 (Table S4). qRT-PCR was performed using Bio-Rad IQ5, as described previously^52^. The relative expression of each gene was analyzed using the 2^-ΔΔ*C*t^ method and Bio-Rad IQ5 software^53^. Each sample had three biological replicates and three technical replicates.

### Ethics approval and consent to participate

The current study complies with relevant institutional, national, and international guidelines and legislation for experimental research and field studies on plants (either cultivated or wild), including the collection of plant material.

### Supplementary Information

Supplementary Legends.

Supplementary Figure S1.

Supplementary Table S1.

Supplementary Table S2.

Supplementary Table S3.

Supplementary Table S4.

## Data Availability

The datasets supporting the conclusions of this study are included in this article and its additional files. The mass spectrometry proteomics data have been deposited to the ProteomeXchange Consortium via the PRIDE^54^ partner repository with the dataset identifier PXD045628. The data of this study is currently private and can only be accessed using a single reviewer account that has been created (visit website: https://www.ebi.ac.uk/pride/archive, username: reviewer_pxd045628@ebi.ac.uk, password: f4smJgJn). After logging in, click on the “Review Submission” on the left side, and then click on “PXD045628” to view the data. After the article is published, the data will be made public. Genomic sequences and gene annotation information for pepper were downloaded from http://solgenomics.net/organism/Capsicum_annuum/genome.
