# Supplementary material for: Unveiling molecular mechanisms of pepper resistance to Phytophthora capsici through grafting using iTRAQ-based proteomic analysis
Source: Sci Rep. 2024 Feb 27;14:4789. doi: 10.1038/s41598-024-55596-3 (PMC10899238; doi:10.1038/s41598-024-55596-3)
Supplement: Supplementary file 1 — Supplementary Legends. [file 41598_2024_55596_MOESM1_ESM.docx]

**Supplementary information**

**Figure S1.** Proteomic analysis of ‘ZCM334’ grafted plants and ‘Early Calwonder’ self-rooted grafted plants. (**A**) Basic information statistics of spectra and peptides. (**B**) Distribution of the number of peptides. (**C**) Mass distribution of the predicted proteins. (**D**) Distribution of peptide coverage of the protein sequences.

**Table S1.** Identification of differentially expressed proteins

**Table S2.** List of GO terms for differentially expressed proteins in ER vs. CMR and E_EL vs. CM_EL

**Table S3.** List of KEGG pathways for differentially expressed proteins in ER vs. CMR and E_EL vs. CM_EL

**Table S4.** Primer sequences of genes, encoding differentially expressed proteins, used for qRT-PCR
