# Supplementary material for: Unveiling molecular mechanisms of pepper resistance to Phytophthora capsici through grafting using iTRAQ-based proteomic analysis
Source: Sci Rep. 2024 Feb 27;14:4789. doi: 10.1038/s41598-024-55596-3 (PMC10899238; doi:10.1038/s41598-024-55596-3)
Supplement: Supplementary file 2 — Supplementary Figure S1. [file 41598_2024_55596_MOESM2_ESM.pdf]

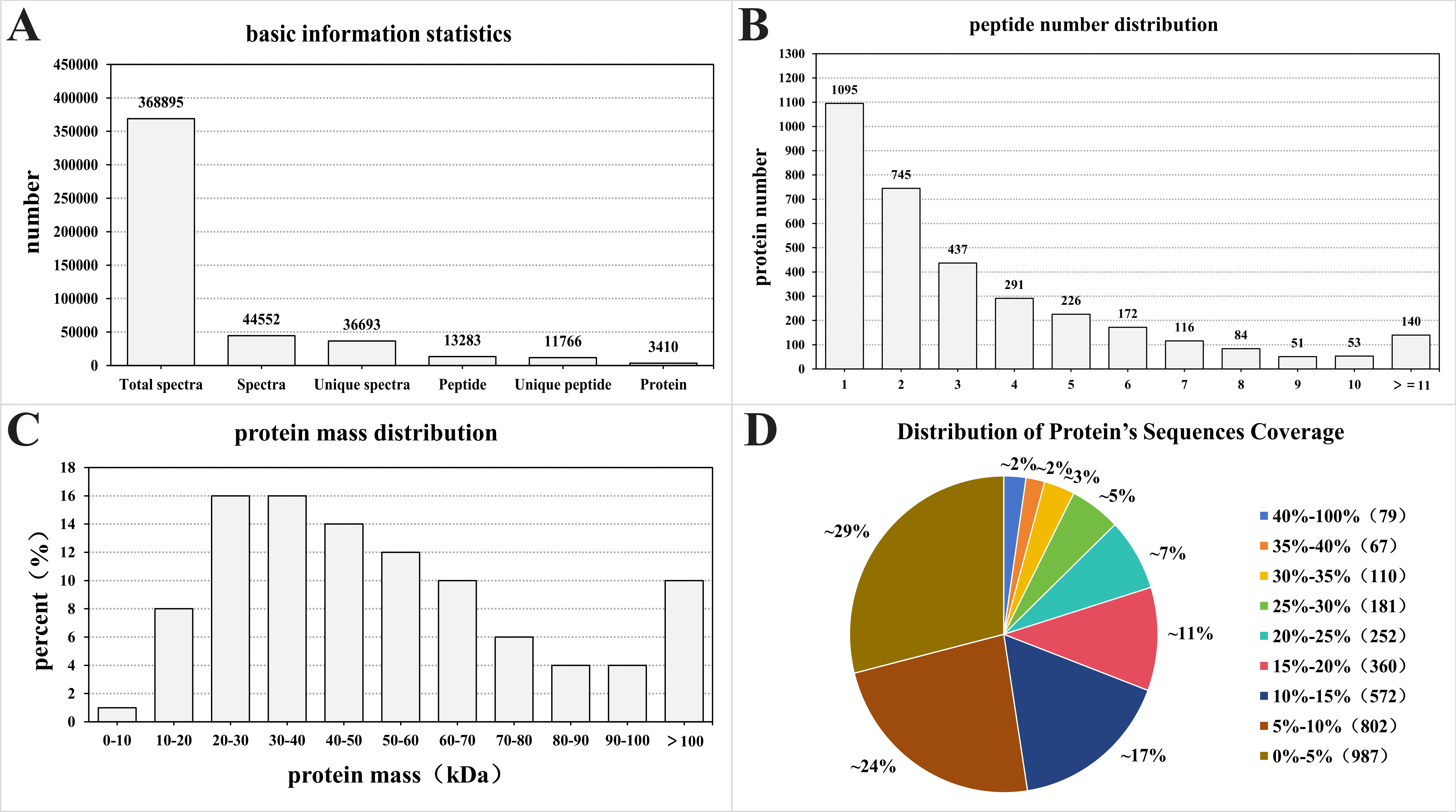

A
basic information statistics
number
450000
400000
350000
300000
250000
200000
150000
100000
50000
0
368895
44552
36693
13283
11766
3410
Total spectra
Spectra
Unique spectra
Peptide
Unique peptide
Protein
B
peptide number distribution
protein number
1300
1200
1100
1000
900
800
700
600
500
400
300
200
100
0
1095
745
437
291
226
172
116
84
51
53
140
1
2
3
4
5
6
7
8
9
10
> = 11
C
protein mass distribution
percent（%）
18
16
14
12
10
8
6
4
2
0
0-10
10-20
20-30
30-40
40-50
50-60
60-70
70-80
80-90
90-100
> 100
protein mass（kDa）
D
Distribution of Protein's Sequences Coverage
~2%
~2%
~3%
~5%
~7%
~11%
~17%
~24%
~29%
40%-100%（79）
35%-40%（67）
30%-35%（110）
25%-30%（181）
20%-25%（252）
15%-20%（360）
10%-15%（572）
5%-10%（802）
0%-5%（987）
